# Preparation and application of single-atom nanozymes in oncology: a review

**DOI:** 10.3389/fchem.2024.1442689

**Published:** 2024-08-12

**Authors:** Huiyuan Liang, Yijie Xian, Xujing Wang

**Affiliations:** ^1^ School of Chemistry and Chemical Engineering, Guangzhou University, Guangzhou, China; ^2^ School of Life Sciences, Sun Yat-sen University, Guangzhou, China

**Keywords:** single-atom nanozymes, nanomaterials, biomedical, cancer treatment, first-principle

## Abstract

Single-atom nanozymes (SAzymes) represent a cutting-edge advancement in nanomaterials, merging the high catalytic efficiency of natural enzymes with the benefits of atomic economy. Traditionally, natural enzymes exhibit high specificity and efficiency, but their stability are limited by environmental conditions and production costs. Here we show that SAzymes, with their large specific surface area and high atomic utilization, achieve superior catalytic activity. However, their high dispersibility poses stability challenges. Our review focuses on recent structural and preparative advancements aimed at enhancing the catalytic specificity and stability of SAzymes. Compared to previous nanozymes, SAzymes demonstrate significantly improved performance in biomedical applications, particularly in tumor medicine. This progress positions SAzymes as a promising tool for future cancer treatment strategies, integrating the robustness of inorganic materials with the specificity of biological systems. The development and application of SAzymes could revolutionize the field of biocatalysis, offering a stable, cost-effective alternative to natural enzymes.

## 1 Introduction

The specific structure of enzymes determines their high catalytic efficiency and catalytic specificity. Most natural enzymes, which are proteins produced by living organisms (Jiang and Liang, 2021), have activity that is highly dependent on specific environmental conditions such as pH and temperature, making them susceptible to deactivation. In addition, natural enzymes have high production costs, low yields, and are difficult to apply on a large scale in industrial production due to their limited lifespan and difficulty in recycling. To overcome these limitations, scientists have been exploring alternative solutions, hoping to find an artificial enzyme with high catalytic efficiency, strong stability, low cost, and simple preparation process. Nanozymes have therefore been proposed. Nanozymes encompass a variety of types, ranging from the metal oxide nanozymes initially discovered by Gao et al. (2007), to later developments in metal organic frameworks (Li et al., 2020), and more recently, to polypeptide protein aggregates. Unlike natural enzymes, which derive their activity from protein folding, nanozymes are characterized by their assembly into nanostructures, leading to distinct mechanisms of activity. Erkang Wang defined nanozymes as enzymes that still exhibit high catalytic activity over a wide temperature range and pH range (Nanozymes, 2018). Its essence is a nanomaterial, and the nanomaterial effect enables it to exhibit the efficient catalytic activity of natural enzymes.

**FIGURE 1:**
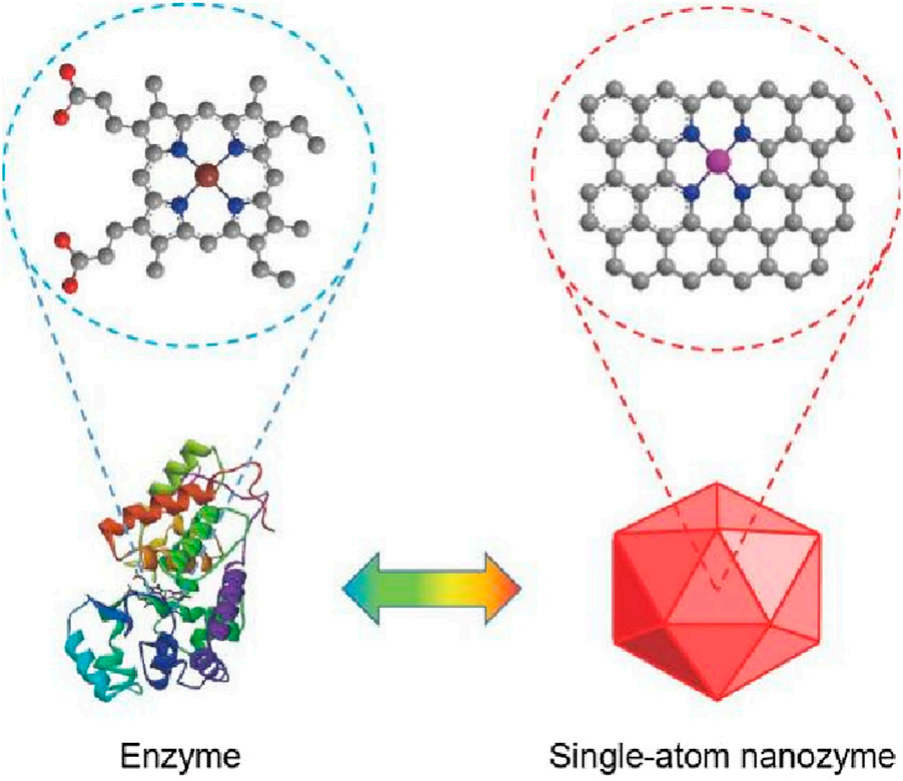
Similar catalytic activity between enzymes and single-atom nanozymes (Jiang and Liang, 2021).

Despite the advantages of nanozymes, their catalytic activity often falls short of natural enzymes due to a low density of active centers (Sun et al., 2022; Xiao et al., 2023). In recent years, Single-atom catalysts (SACs) was prepared, a type of catalyst uniformly disperses single atoms on the carrier with a certain distance. This certain distance endow SACs with high catalytic activity (Figure 1). In addition, as research on SACs deepens, the concept of single-atom nanozymes (SAzymes) emerged accordingly (Xie et al., 2023). The metal centers of these SAzymes are highly dispersed (Cai et al., 2023), and the catalytic sites are uniformly distributed as single atoms, thus greatly improving the atomic utilization efficiency of the active center (Peng et al., 2024). Its catalytic activity is 10–100 times that of traditional nanozymes, almost reaching the level of natural enzymes (Pei et al., 2020). Designing active sites with atomic-level distribution on the carrier can maximize atomic regulation, thereby bridging the gap in efficiency, selectivity, and catalytic performance between natural enzymes and SAzymes (Xu et al., 2021). Due to the high catalytic activity of SAzymes, they can effectively respond to the acidic and hypoxic microenvironment of tumors. By utilizing abundant substrates, such as hydrogen peroxide (H_2_O_2_), SAzymes generate reactive oxygen species (ROS) that induce apoptosis in cancer cells and deplete the reducing agents or nutrients necessary for their survival. This process ultimately facilitates effective antitumor therapies (Guo et al., 2024). Additionally, the hypoxic microenvironment not only hinders the antitumor activity of related immune cells but also fosters the proliferation and metastasis of tumor cells. The modulation of the tumor hypoxic microenvironment by SAzymes can enhance the oxygen content at tumor sites (Jiang et al., 2024).

This article summarizes the structural characteristics and preparation methods of SAzymes in recent years and introduces the latest progress of SAzymes in tumor medicine such as biosensing and anti-tumor. SAzymes precisely construct and regulate the active center at the atomic level (Shen et al., 2024), and their small size at the micro nano level endows them with high specific surface area and high atomic utilization, thus possessing efficient catalytic activity comparable to proteases. By carefully regulating the active center of SAzymes to resemble that of natural enzymes, their catalytic activity can be increased (Xie et al., 2020; Chen et al., 2023a; Wang et al., 2023a; Yang et al., 2023). However, this atomic scale also poses great challenges to the structural regulation of SAzymes (Bhalothia et al., 2024). Meanwhile, SAzymes lack a certain degree of specificity. To address this issue, organic frameworks are generally used to regulate the local coordination structure of the central atom.

**FIGURE 2:**
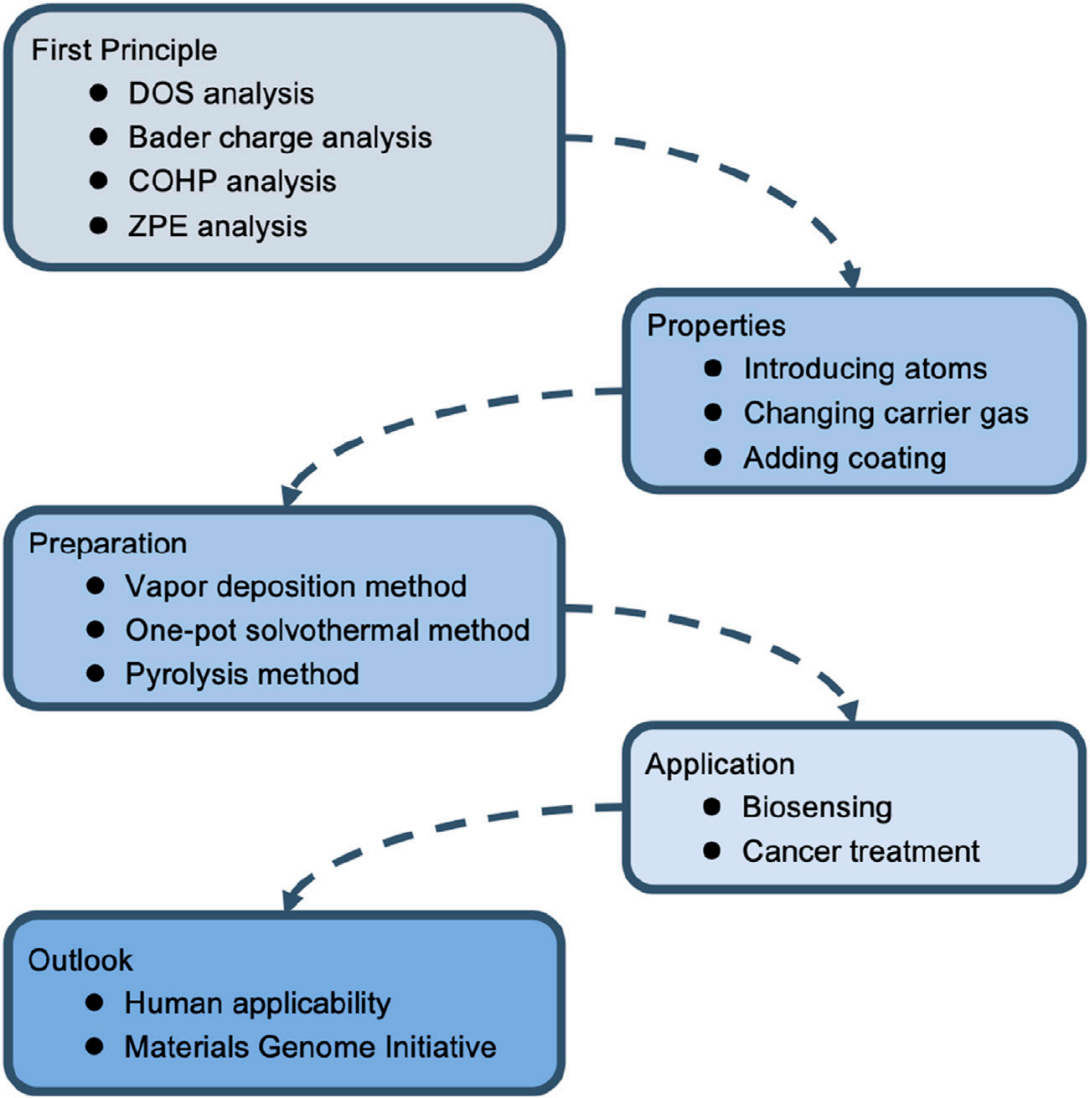
The structure of this review.

SAzymes have been widely used in the field of biomedicine, especially in the field of oncology; The research mainly focuses on its catalytic performance, and there is relatively little research on whether it will bring side effects; In addition, nowadays, what widely used are non-precious metals such as, Fe, Ni, this limits the types of materials available, and future researches need to focus on the integration and application of the Materials Genome Initiative (Figure 2).

## 2 The foundation of metal organic framework type single-atom nanozymes

In the field of catalysis, first principles are usually used to design SAzymes from a theoretical perspective. At present, most SAzymes belong to metal organic framework type SAzymes. Isolated metal atoms tend to aggregate, so SAzymes are generally difficult to exhibit the desired effect. The solution is to disperse SAzymes in some frameworks to increase the interaction between individual metal atoms and substrates, in order to solve the problem of metal single atom clusters (Chen et al., 2021; Bhalothia et al., 2024). Carbon based materials have a large surface area and strong interactions with metal centers, making them the first choice for constructing SAzymes (Wan et al., 2023). This organic framework can also interact with metal central ions to affect the local coordination structure of active sites, thereby altering its catalytic performance (Xie et al., 2022). In order to improve the dispersion of atoms, hydrocarbons with a network structure are generally chosen, which act like cages separating the centers of metal atoms. Commonly used compounds with hydrocarbon network structures include zeolitic imidazolic acid skeleton-8 (ZIF-8), carbon dots (CDs), and ZIF-67. In addition, the higher dispersion of single atoms increases their specific surface area, thus increasing their catalytic activity (Wang and Zhou, 2021). They have been proven to function like natural enzymes (Zhang et al., 2022; Zhu et al., 2023a). The common preparation methods for SAzymes currently include vapor deposition method, one-pot solvothermal method, pyrolysis method (Hua et al., 2024).

### 2.1 The application of first principles calculations in the design of single-atom nanozymes

First principles calculations, especially those based on density functional theory (DFT), have become one of the core tools in modern catalytic science. These calculation methods are based on basic physical laws and do not rely on any empirical parameters, understanding and predicting the structure and reaction mechanism of catalysts from a microscopic level. By accurately calculating the electronic structure, density functional theory has helped researchers reveal the properties of catalyst active sites, which are the most critical parts of catalytic reactions.

In the computational research of catalysts, the determination of active sites is crucial. Evaluation methods include several key calculations and analyses. Calculating adsorption energy is used to determine the stability of molecules on the catalyst surface. Density of States (DOS, can be used to evaluate orbital components) analysis is then employed to study the distribution and energy level structure of electronic states, which includes calculating the d-band center. Bader charge analysis (can be used to qualitatively calculate valence) provides an accurate calculation of charge distribution, helping to understand the relationship between charge migration and catalytic activity. Crystal Orbital Hamiltonian Population (COHP) analysis is used to identify interactions between molecular orbitals. Zero-point energy correction (ZPE, makes the evaluation of reaction energy more accurate) is applied to evaluate the impact of quantum mechanical effects on catalytic activity.

Bolong Xu et al. (2022) used DOS analysis and found that compared to FeN_4_ SAzyme, the DOS of FeN_5_ SAzyme is significantly higher near the Fermi level, and several new hybrid electronic states appear, indicating a stronger interaction between FeN_5_ SAzyme and H_2_O_2_. Wang et al. (2022b) used adsorption energy, Bader charge, COHP analysis and DOS analysis to illustrate a better catalytic performance of MnSA-N_3_-C compared to MnSA-N_4_-C. The exploration of computational methods has never stopped. Due to the fact that the d-band center theory is often more suitable for metal atoms in clusters with close distances. Their energy levels overlap, so strong hybridization can be observed in a wide energy range on DOS. However, for SAzymes, metal atoms are far apart, and energy levels split under the action of crystal fields, corresponding to different frontier orbitals. For example, for Au atomic catalysts, there have been numerous studies discussing their catalytic activity (Camellone and Fabris, 2009; Zhou et al., 2018), but no consensus has been reached. However, Fu et al. (2020) used the frontier orbital method to analyze the orbital overlap between Au atoms and substrates, and successfully obtained an accurate dominant configuration of Au atom catalysts in the absence of a contradiction between d-band center theory and adsorption energy. According to the actual situation, the catalytic mechanism of SAzymes can be more comprehensively elucidated by combining multiple theoretical calculation methods. In addition to calculations in conventional environments, simulating catalytic reaction conditions in more complex environments (such as temperature, pressure, and chemical environment) makes catalytic systems more efficient. Zou et al. (2020) considered the influence of pressure on adsorption energy in their study of N_2_ reduction, designed more specific reaction pathways, and provided more specific activation energy values, which is of great significance for catalyst selection.

Once the application of first principles methods in catalysis is established, it is essential to design experimental methods to identify the necessary transition states. The introduction of the Materials Genome Initiative can be seen as an innovative solution to the current limited development in the field of SAzymes. By systematically modeling potential catalyst skeletons and replacing their central metal atoms, this strategy can generate a wide variety of different catalyst structures. On this basis, utilizing adsorption module in advanced materials science tools such as pymatgen and ase can efficiently generate multiple adsorption site models (Ong et al., 2013; Larsen et al., 2017). Subsequently, minimizing the energy of these models can not only find the ground state structure of the system, but also conduct further adsorption energy analysis. This method effectively combines computational chemistry and experimental chemistry. The allocation of experimental resources can be optimized with large-scale pre-screening before experimental operations.

**FIGURE 3:**
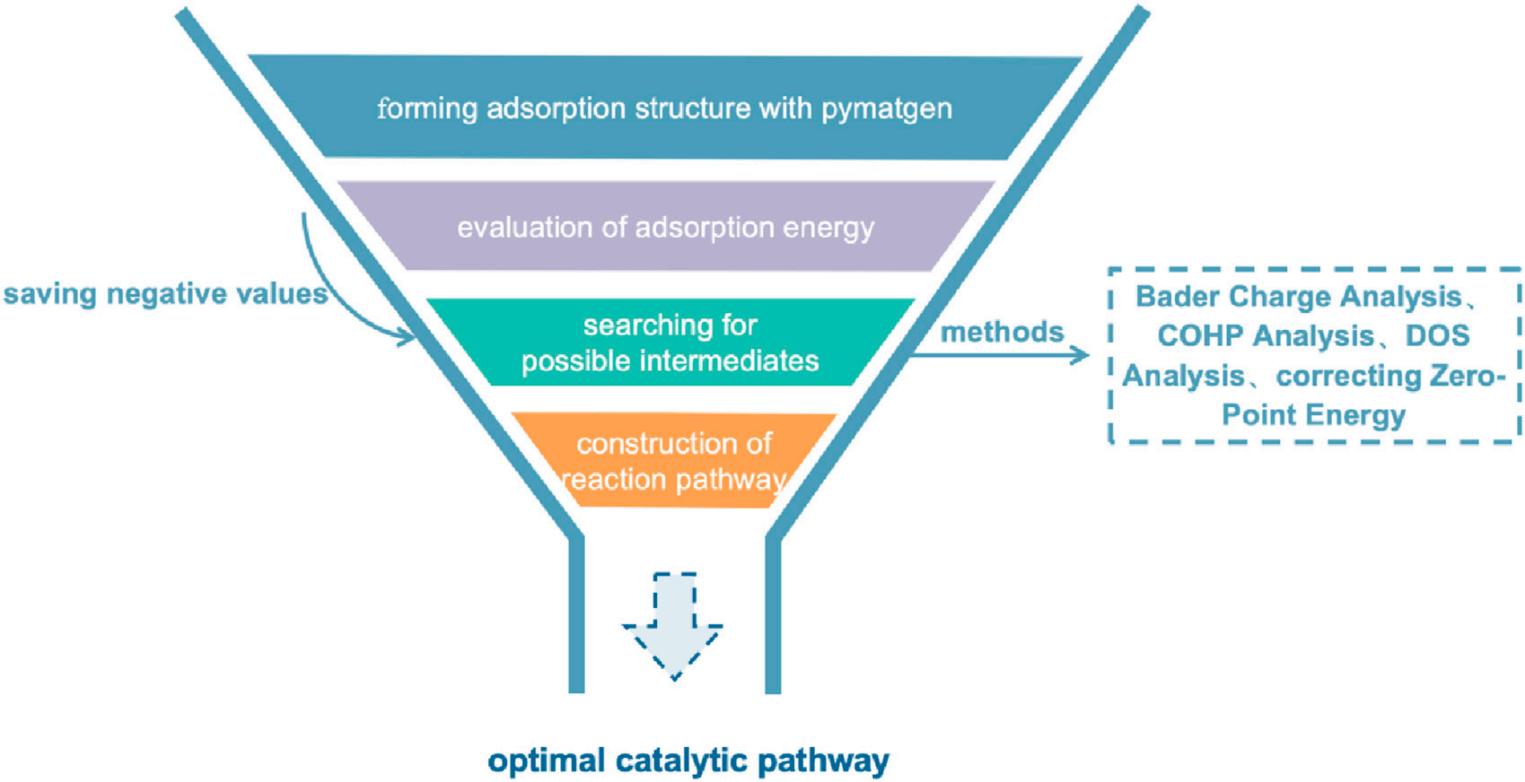
Screening an optimal catalytic pathway.

Following the preliminary evaluation of active sites, first principles calculation methods such as Bader charge analysis, COHP, and DOS analysis can be employed to meticulously compare the catalytic performance of different SAzymes with the same reaction substrate. Bader charge analysis is mainly used to quantitatively understand the degree of charge transfer between metal atoms and reaction substrates; COHP analysis provides a method for evaluating the strength of chemical bonds between metal atoms and reaction substrates. By analyzing the contributions of each energy level in the energy band, we can understand the covalent and ionic properties of chemical bonds, thereby predicting the tendency of chemical bond breakage and formation in chemical reactions. DOS analysis focuses on the hybridization degree of orbitals between metal atoms and reaction substrates, especially in transition metal catalysts, where the p-d hybridization phenomenon between their d-orbitals and the p-orbitals of reaction substrates is particularly important. For catalysts containing transition metals with d-orbitals, magnetic exchange mechanisms [including direct exchange (Van Vleck, 1953), super exchange (Anderson, 1959) and double exchange (De Gennes, 1960)] are also dynamically affect the progress of catalytic reactions. These mechanisms affect electrons’ control towards the reaction pathway by altering the spin arrangement of electrons. This complex interaction is crucial in catalytic science as it can determine the stability and activity of catalysts in different reaction environments (Figure 3).

### 2.2 Structure and properties

The essence of SAzymes is nanomaterial. The activity and specificity of nanomaterials is different because of their different structure, so the effectiveness of their application in tumor treatment is also different. Reasonably designing the types of central atoms and ligands can effectively regulate DOS and d-orbital states of central atoms, optimize the adsorption free energy of substrate and thus efficiently promote its conversion (Wan et al., 2023).

**FIGURE 4:**
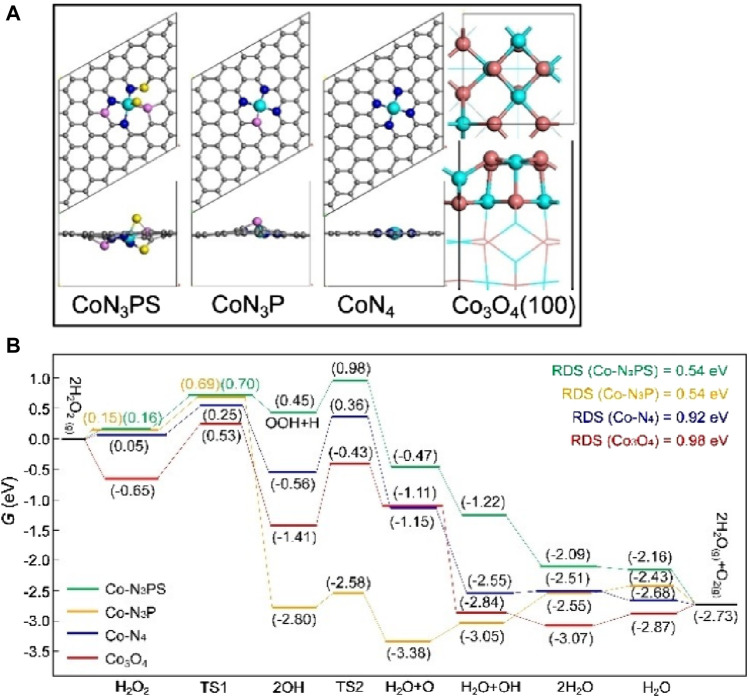
(Chen et al., 2023a) **(A)** The top and side view structures of Co-N_3_PS, Co-N_3_P, CoN_4_ and Co_3_O_4_. **(B)** Under neutral conditions, free energy diagrams of possible catalytic processes for Co-N_3_PS, Co-N_3_P, CoN_4_ and Co_3_O_4_ Color code: Co (green), C (gray), N (blue), P (pink), S (yellow), O (orange).

After selecting the central atom, the common method is to change the ligand. This alters the charge distribution and coordination environment, enabling precise regulation of the active sites of SAzymes by modifying the local coordination structure (Figure 4A). This can better simulate the active sites and spatial configurations of natural enzymes and improve their catalytic activity. The more similar the structure of this complex is to that of a natural enzyme, the higher its catalytic activity. By examining the free energy diagram of SAzymes, the catalytic activity of the catalyst can be roughly determined (Chen et al., 2023a) (Figure 4B).

**FIGURE 5:**
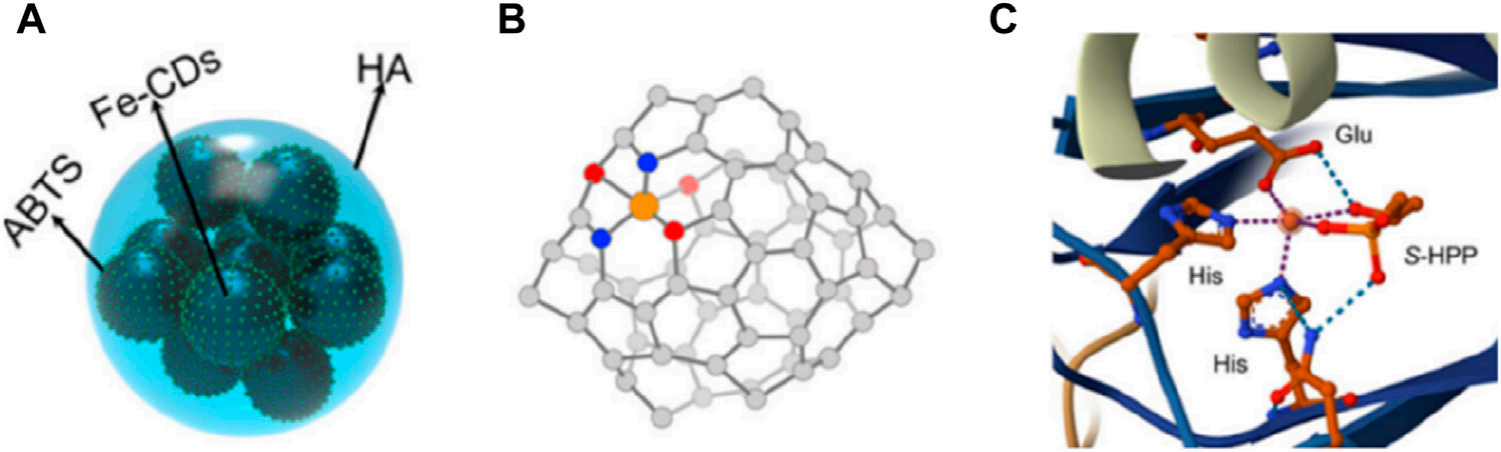
**(A)** The model of Fe-CDs/ABTS@HA. **(B)** The catalytic active center of Fe-CDs/ABTS@HA. **(C)** The catalytic active center of HppE.

CDs are a type of carbon based material with ultra small size and abundant surface functional groups, which have significant advantages in riveting and exposing active metal sites. Therefore, the rich oxygen-containing groups on carbon dots can be utilized to regulate the local coordination structure by interacting with metal centers (Zhang et al., 2023). Yang et al. (2023) utilized CDs as organic frameworks to adjust the active centers of SAzymes, resulting in a morphology similar to that of peroxidase (POD). Due to the coordination between CDs and Fe^3+^, atomically dispersed Fe (II) can be stabilized by oxygen atoms, forming Fe-O_3_N_2_. The active site structure of iron SAzymes (Fe-O_3_N_2_) is similar to that of (S) -2-hydroxypropyl and 1-phosphonate (S-HPP) epoxidase (HppE), a typical natural non heme iron based peroxidase (Figure 5C). This iron SAzymes have relatively saturated single iron atom sites, so its POD-like activity is 750 units/mg, much higher than the traditional SAzymes of Fe-N_4_ and Fe-N_3_P with planar structures (316 units/mg), and natural Horseradish Peroxidase, (HRP) (504 units/mg).

The interaction between sulfur atoms and metal centers also makes it possible to regulate local coordination structures. In 2024, Wendong Liu et al. (2024) utilized the doping of S atoms to disrupt the symmetric charge distribution around the Fe single atom, and designed Fe-S/N-C SAzymes (coordination structure is Fe-N_3_S_1_) with asymmetric charge distribution. Bader charge analysis shows that the charge of iron atoms in the asymmetric coordination structure Fe-N_3_S_1_ is lower than that in the symmetric coordination structure Fe-N_4_. This enhanced electron localization can optimize the adsorption energy of oxygen-containing substances such as H_2_O_2_, endowing Fe-S/N-C SAzymes with strong POD-like activity (Xu et al., 2023). Based on measurements and calculations using the Arrhenius equation, the activation energies (E_a_) for H_2_O_2_ activation catalyzed by Fe-S/N-C and Fe-N-C are 10.31 kJ/mol and 18.12 kJ/mol, respectively. According to the electron spin resonance (ESR) spectrum, Fe-S/N-C exhibits a higher intensity (f OH dimethylpyridine nitrate oxide) DMPO signal, confirming its superior POD-like activity. The rate constant of Fe-S/N-C consuming glutathione (GSH) is 0.46 min^−1^, which is higher than the rate constant of Fe-N-C consuming GSH (0.04 min^−1^), indicating that Fe-S/N-C has higher GSH-like activity. In 2024, Xu et al. (2024) designed a copper based SAzyme (Cu-N/S-C SAzymes) using sulfur engineering technology, which introduces S atoms into the catalyst to redistribute the electrons of copper atoms, presenting an asymmetric distribution. The introduction of S atoms enhances the electron transfer between the active center Cu-N_1_S_2_ and H_2_O_2_, and also promotes the adsorption and activation process of H_2_O_2_, resulting in better generation of OH. Cu-N/S-C SAzymes exhibiting higher catalase-like(CAT-like) activity compared to Cu-N-C SAzymes with Cu-N_3_ sites, the affinity of Cu-N/S-C SAzymes for hydrogen peroxide substrate increased by 13.8 times, and the catalytic efficiency increased by 65.2 times. Meanwhile, Cu-N/S-C SAzymes have a high photothermal conversion efficiency (31.7%) and good photothermal cycling stability, which makes them potential as photothermal agents in tumor treatment.

Changing the type of carrier gas can alter the valence state of the central atom, thereby altering its local coordination environment. In 2022, Wang et al. (2022a) utilizes the abundant and adjustable defects on the surface of TiO_2_ to fine tune the local coordination environment of the active site (Co atom). The XPS results showed that cobalt based SAzymes (Co/A-TiO_2_ SAzymes) calcined in air had a lower Co^2+^/Co^3+^ ratio than cobalt based single atom nanozymes (Co/N-TiO_2_ SAzymes) calcined in nitrogen. Theoretical studies have shown that the lower the charge of Co, the higher the activity of catalyzing the decomposition of H_2_O_2_ into O_2_; The Michaelis Menten kinetic experiment results indicate that at pH 6.5, the Michaelis Menten constant (K_m_) of Co/A TiO_2_ catalyzing H_2_O_2_ is 1.95 mM, and the maximum initial velocity (V_max_) is 1.22 × 10^−6^ M s^−1^, which is superior to most single atom nanozymes reported in the past (their V_max_ value is one quantity higher than previous systems). Therefore, Co/A-TiO_2_ SAzyme calcined in air has higher catalytic activity.

In order to enhance the practicality of single atom nanozymes, coatings can be added on the surface of SAzymes to give them some certain functions. In order to improve the colloidal stability and prolong the blood circulation time of CDs doped iron SAzymes (Fe-CDs), Qingyuan Yang et al. (2023) encapsulated ABTS on their surface and coated it with a layer of HA to give them a typical quasi spherical shape (Figures 5A, B). In 2024, Liu et al. (2024) used macrophage membranes to encapsulate Fe-S/N-C SAzymes in order to endow them with targeting ability. Vascular cell adhesion protein 1(VCAM-1) expressed on the surface of cancer cells, can bind to α_4_ and β_1_ integrins overexpressed on the surface of macrophages, thereby achieving effective tumor targeting. Long et al. (2024) utilized the high dispersibility of CDs in water or buffer solutions to prepare an iron SAzyme with good water solubility and easy target sensing in water.

### 2.3 Preparation methods

#### 2.3.1 Vapor deposition method

**FIGURE 6:**
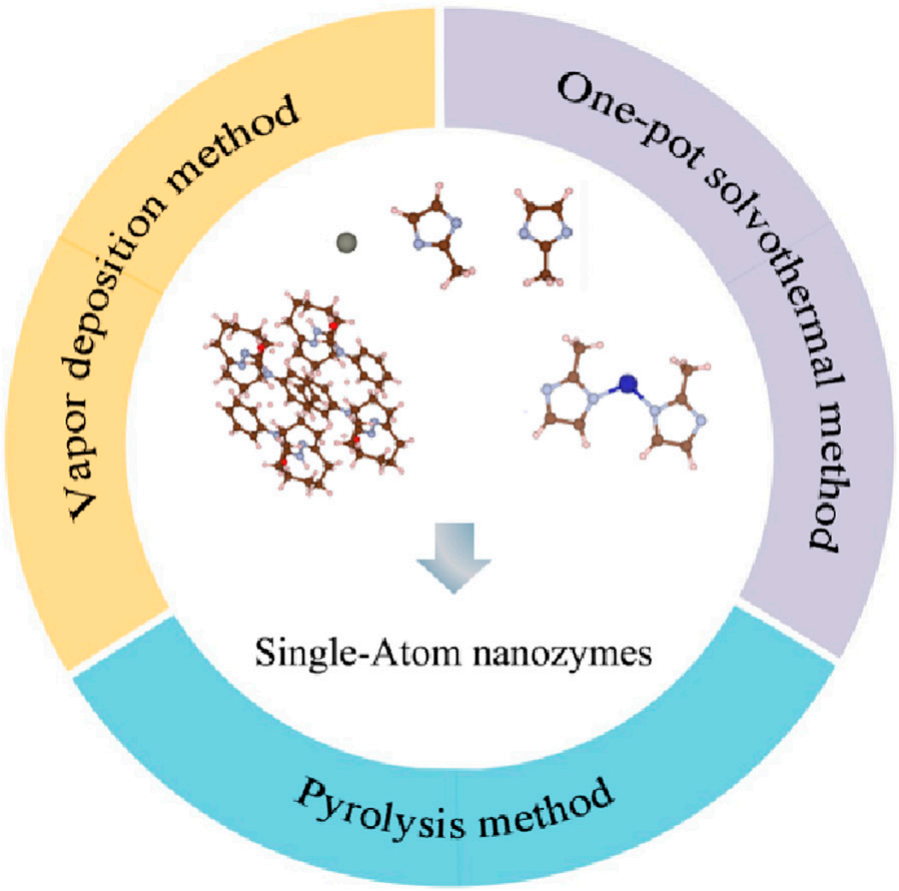
Different preparation methods for SAzymes.

**FIGURE 7:**
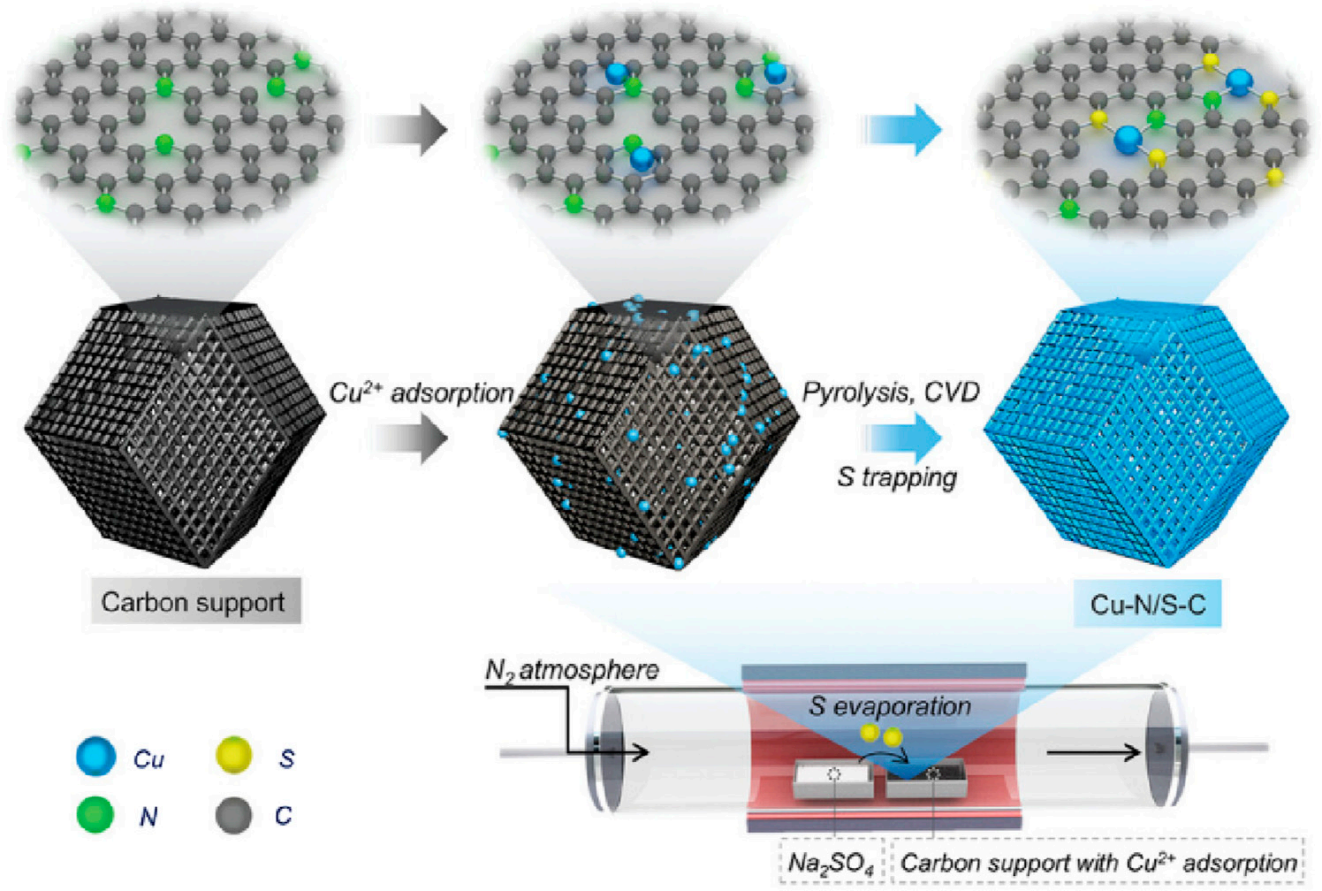
(Xu et al., 2024) The preparation of Cu‐N/S‐C.

In the process of vapor deposition method, the reactants are usually heated at high temperatures, diluted with rare gas or N_2_ to dilute the vapor phase composition, and deposited in a single atom dispersed form on the substrate surface Figure 6. Xu et al. (2024) used silicon dioxide to protect the pyrolysis of ZIF-8 under nitrogen atmosphere at 1,000°C, obtaining a carbon carrier. After adsorbing copper ions onto carbon carriers, Xu placed them at the lower airflow outlet of the tube furnace, placed sodium sulfate powder at the upper airflow outlet of the tube furnace. After pyrolyzing, Cu-N/S-C SAzyme whose active center is Cu-N_1_S_2_ can be obtained (Figure 7). Qin et al. (2022) used atomic deposition technology (ALD), a modified chemical vapor deposition technique to mix 45 mL DMF, 135 mL IPA, and 4.5 mL C_16_H_36_O_4_Ti for a reaction. After two high-temperature treatments, TiO_2_ support was obtained. Introducing ultra-high purity N_2_ to a self-designed ALD reactor, they prepared xFeO_x_/TiO_2_ nanozyme. Using the ALD ultra-thin modification strategy, xFeO_x_/TiO_2_ nanozymes were used as carriers to load FeO_x_. As the number of TiO_2_ ALD cycles increased, highly dispersed iron-based SAzymes limited to porous TiO_2_ nanospheres were obtained. After calcination and reduction treatment, TiO_2_ can be transformed from an amorphous form to a crystalline form, resulting in iron-based SAzymes with twice the activity.

#### 2.3.2 One-pot solvothermal method

According to the low solubility of salts in organic solvents, one-pot solvothermal method can be used to reduce the collision probability between particles, resulting in highly dispersed SAzymes. Chen et al. (2023b) utilizes the condensation reaction between formamide molecules during the solution heating process (condensation of primary amines and active carbonyl compounds makes it easy to synthesize Schiff bases), coordination of Schiff bases and Fe ions, and simultaneous occurrence of carbonization to design a Schiff bases-derived Fe-NC-x SAzymes. They adopted one-pot solvothermal method, using different concentrations of iron ions and formamide as raw materials. After high-temperature treatment, they were centrifuged, washed, and dried to obtain Fe-NC-x SAzymes (x reflects the relative content of iron ions and formamide). This synthesis strategy has a certain universality, which can be extended to prepare SAzymes with different metal atomic centers, such as Cu Mn, Co, Zn, and Ni. Bo Wang et al. (2022b) synthesized manganese nitrogen co-doped carbon materials (f-MnNC) containing monodisperse Mn-N_4_ sites using MnCl_2_ and formamide as precursors through one-pot solvothermal method at 180°C for 12 h. This manganese single-atom nanozyme has efficient peroxidase-like activity. Lin et al. (2020) add 0.2 g of citric acid and a certain amount of sodium chlorophyllin copper salt (CuCh) to 4.0 mL of deionized water, and add 1 mL of 0.6 mg/mL polyoxyethanolamine to it. After mixing evenly, add 4.0 mL of absolute ethanol. After heating the mixed solution at 200°C for 10 h, cooling, filtering, and dialysis, Cu-N-C SAzymes can be obtained. Zhu et al. (2023) prepared TiO_2_ nanotubes in a 1.0 wt% hydrofluoric acid aqueous solution under a working voltage of 20 V for 30 min. After removing the remaining electrolyte in deionized water, it annealed at 450°C for 2 h and labeled with TNT. Then, Fe-NC nanozymes were prepared on the surfaces of Ti and TNT, using 100 mL of formamide solution containing 10 mM FeCl_3_ as the solvent.

#### 2.3.3 Pyrolysis method

**FIGURE 8:**
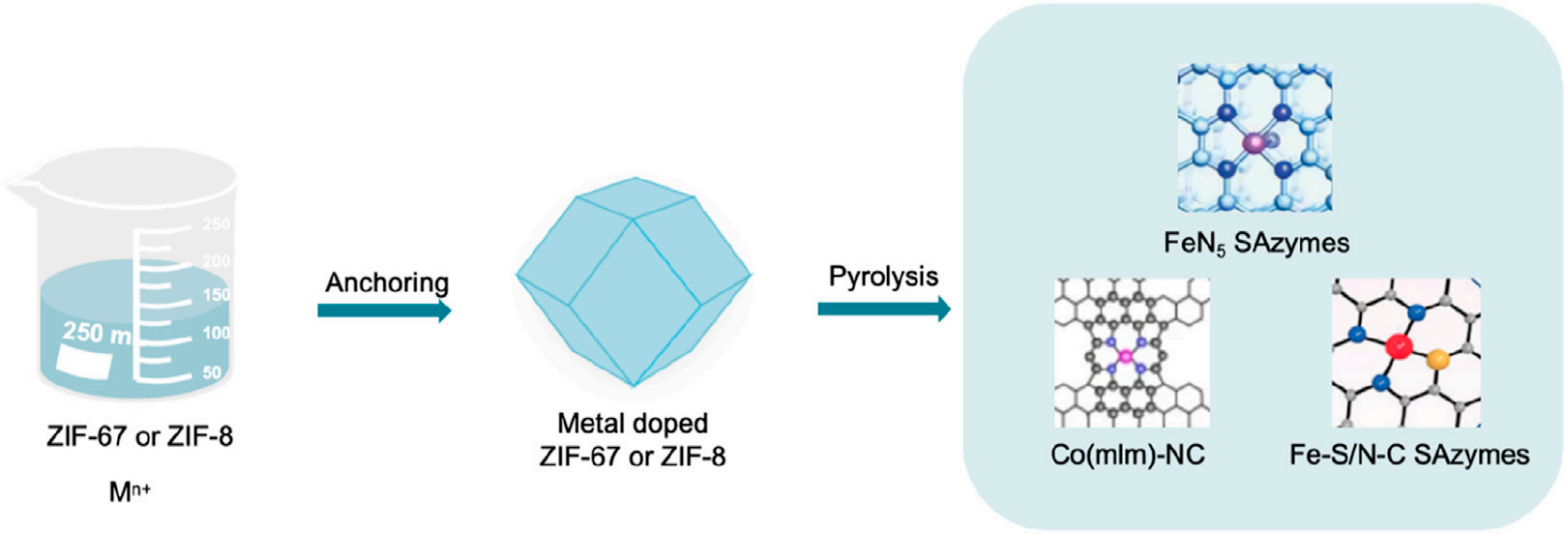
Key steps of the pyrolysis method and the structure of SAzymes prepared by pyrolysis (Xie et al., 2020; Xu et al., 2022; Liu et al., 2024).

The pyrolysis method mainly includes two key steps. Fixing metal ions in organic frameworks and evaporating some atoms at high temperatures to anchor isolated metal centers within the substrates (Figure 8). Sun et al. (2022) prepared precursors, bimetallic Zn/Co metal organic frameworks (ZIF-67 doped with zinc) (Cao et al., 2020), using Zn(NO_3_)_2_·6H_2_O, Co(NO_3_)_2_·6H_2_O and 2-MI. Subsequently, it was pyrolyzed in an argon atmosphere and cooled to obtain Co-N-C SAzymes, a material with Co single atoms uniformly dispersed on nitrogen doped porous carbon. In 2022, Bolong Xu et al. (2022) employed a two-step pyrolysis method mediated by melamine, utilizing zeolite imidazole framework-8 (ZIF-8) as a precursor and coating its surface with SiO_2_. Initially, pyrolysis was conducted at a temperature of 1,000°C to obtain a monodisperse single carbon carrier, followed by the deposition of Fe^3+^ onto its surface. Subsequently, it was mixed with melamine, a nitrogen-rich precursor. Ultimately, an iron-based SAzyme with a five-coordinated (FeN_5_) structure was prepared via pyrolysis at 900°C.

In 2023, Yang et al. (2023) partially carbonized the precursor Fe (III)–tannic acid (TA), stabilizing and dispersing individual Fe(II) by the phenolic hydroxyl groups of Fe (III)–TA and using the dehydration network as a three-dimensional carbon framework to form monodisperse quasi spherical nanoparticles (Fe-CDs/ABTS@HA). In 2024, Wang et al. (2022a) uniformly dispersed Fe doped zeolite imidazolium salt framework-8 (Hemin/ZIF-8) in methanol solution, and then underwent pyrolysis at 920°C when it was partially carbonized and evolved into an asymmetric Fe-N_3_S_1_ portion (represented as Fe-S/N-C). The single atom catalyst with this Fe-N_3_S_1_ active center is Fe-S/N-C SAzymes. Xie et al. (2020) first added Co(acac)_3_(acac, acetylpropionat), Zn(NO_3_)_2_·6H_2_O and 2-methylimidazole (mlm) in a methanol solution to encapsulate Co(acac)_3_ in ZIF-8 micropores. Then, it was subjected to a solvothermal treatment at 140°C for 4 h, which can be converted into Co(mlm)_4_ through ligand exchange. Pyrolysis can directly convert it into a SAzyme named Co(mlm)-NC, with CoN_x_ sites atomically dispersed in porous carbon.

## 3 Applications of single-atom nanozymes in oncology

**FIGURE 9:**
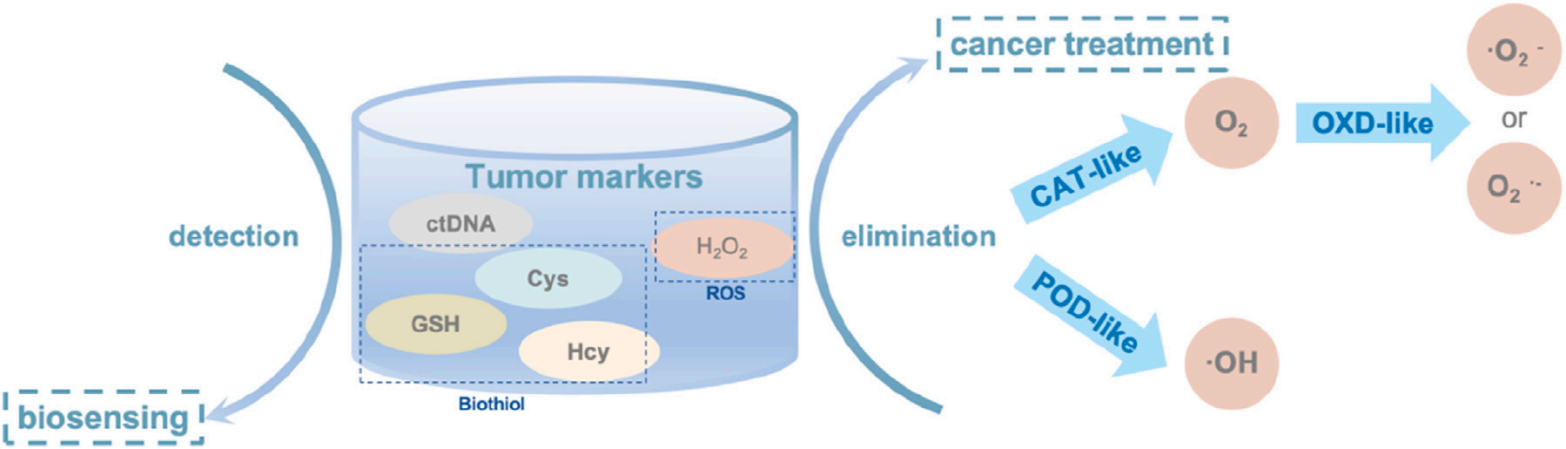
The main tumor markers and their applications in oncology, the elimination mechanism of H_2_O_2_ in oncology.

In oncology, SAzymes are primarily used to mimic natural enzymes for detecting the concentration of biomolecules, thereby detecting the presence of tumor cells Figure 9. They also simulate oxidoreductases to promote the transformation of toxic substances in the body, thus achieving cancer treatment purposes.

### 3.1 Biosensing

Biosensor technology based on SAzymes has found extensive applications in disease diagnosis due to its high active site density and atomic utilization efficiency, which results in faster reaction rates, higher sensitivity, and accuracy (Chen et al., 2023c). Currently, SAzymes are primarily used in cancer screening (Liang et al., 2023). Canceration cells typically produces various cancer markers, and employing SAzymes as biosensors enables more efficient and rapid detection of these markers, allowing for earlier intervention and treatment of cancer patients. The main cancer markers currently detected include biothiols and circulating tumor DNA (ctDNA) (Wang et al., 2024).

#### 3.1.1 Detection of biothiol

Biothiols primarily refer to GSH and cysteine (Cys). Biothiols are abnormally expressed in cancer cells, resulting in higher concentrations in cancer cells compared to normal cells. Therefore, they can be used as tumor markers for cancer screening in clinical practice. Sun et al. (2022) have developed a Co-N-C SAzyme based on the acid-base pairing theory for detecting biothiol levels. This SAzyme exhibits strong coordination between the Co atoms and the S atoms of biothiols, enabling specific binding. Using a spectrophotometer, a working curve can be plotted to determine the concentration of solutions containing cancer markers (biothiols). Sun conducted five repeated measurements and found no significant differences in the absorption rates of GSH/Cys, indicating that the Co-N-C SAzyme has good reproducibility as a biothiol sensor. When other amino acids, with concentrations ten times lower than those of GSH or Cys, were introduced, the detected absorbance was significantly lower than that of GSH or Cys, demonstrating the specificity of the Co-N-C SAzyme. The detection limits for GSH and Cys were 0.07 µM and 0.06 µM, respectively, indicating the high sensitivity of the SAzyme. Therefore, SAzyme have promising applications in the field of biosensing.

#### 3.1.2 Detection of ctDNA

When somatic cells in a healthy human body undergo apoptosis or necrosis, DNA can be released and enter the bloodstream. This type of DNA is called cell free DNA (cfDNA). For cancer patients, the DNA released during the canceration of their cells is called ctDNA (Sakaeda and Naito, 2021). Cancer screening can be conducted by collecting body fluids containing ctDNA (Rossi, 2023). CtDNA carries characteristic changes of tumor cells, such as mutations and methylation (Wen et al., 2022), and can therefore serve as a next-generation tumor marker.

However, ctDNA is relatively unstable in body fluids, with a half-life of approximately 16 min to 2.5 h (Chen et al., 2020). Therefore, finding an efficient method to detect ctDNA levels is particularly important. Long et al. (2024) utilized CDs to prepare a water-soluble iron SAzyme (SA Fe-CDs). The doping of CDs endowed SA Fe-CDs with abundant functional groups, enabling the binding of single strands and the output of large amounts of DNA products, thus improving the sensitivity and specificity of the detection. Using SA Fe-CDs as signal carriers, they developed an electrochemical biosensor that specifically attaches to electrodes. This stable sandwich structure can generate consistent electrochemical signals and further catalytically amplify the signal, resulting in a low detection limit of 1.26 aM. Long set up seven control groups, demonstrating that the biosensor has high reproducibility. The signal output was observed continuously for 28 days, and after 28 days, the signal remained at 89.97% of its original value, indicating high stability. The experiments introducing other types of DNA fragments proved that SA Fe-CDs have high selectivity.

### 3.2 Cancer treatment

Reactive oxygen species (ROS) are byproducts of mitochondrial respiration in organisms, including oxygen-containing radicals as well as peroxides easily to form radicals such as ⋅O^2−^ and H_2_O_2_. ROS are often overexpressed in tumor cells, and the excessive production of ROS can significantly harm cellular components. This includes damaging cell membranes, nucleic acids, and proteins, leading to severe pathological processes such as cancer, diabetes, and sepsis. Overexpression of ROS can also promote the formation of tumor cells. Based on this understanding, a method for cancer therapy involves converting ROS to non-toxic products like O_2_ (Chen et al., 2023b) or toxic radicals (Xu et al., 2022) to target tumor cells. However, the application of natural enzymes in clinical treatment is limited due to their challenging extraction, high cost, low stability, and susceptibility to inactivation. Therefore, SAzymes, which are cost-effective and highly stable, can be used as substitutes.

#### 3.2.1 Converting ROS to non-toxic O_2_

Chen et al. (2023b) discovered that Fe-NC-x SAzymes possess a rich π-conjugated structure, which endows the N-C matrix with high electron affinity. Consequently, the N-C matrix can interact with O^2−^ through π-π stacking, converting it into non-toxic H_2_O_2_ and O_2_.

**FIGURE 10:**
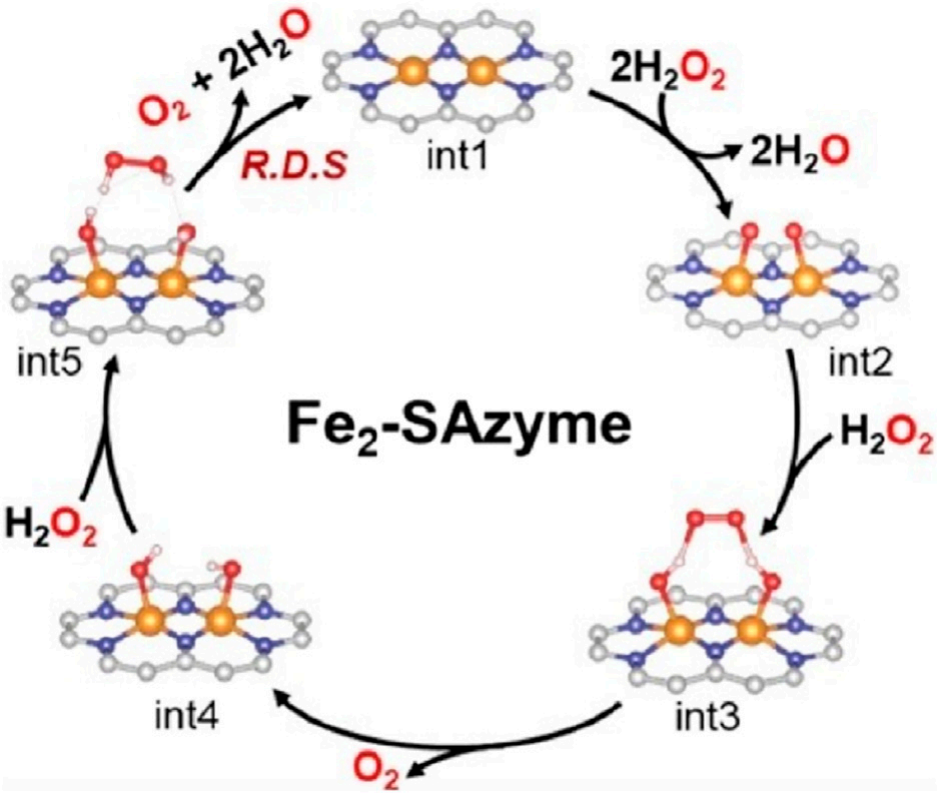
(Zhang et al., 2024) The process of Fe_2_-SAzyme adsorbing H_2_O_2_ and decomposing it into O_2_.

The optimal temperature for Fe-NC-x SAzymes is approximately 40°C, which aligns with the surface temperature of the human body, indicating that this SAzyme can achieve maximum activity when applied in cancer treatment within the human body. Chen conducted stability tests under harsh acidic and alkaline conditions, as well as storage stability tests, demonstrating that its stability is significantly higher than that of natural enzymes. Furthermore, Fe-NC-x SAzymes exhibit good blood compatibility, low cytotoxicity, and specificity in eliminating ROS. Fe-NC SAzymes can decompose H_2_O_2_ to protect cells from oxidative stress. After treatment, the viability of oxidatively stressed cells increased from 1.4% to 80.3%, showing the excellent cell-protective performance of Fe-NC-x SAzymes. The Fe2-SAzyme designed by Zhang et al. (2024) features dual single iron sites, which act as molecular tweezers, effectively capturing H_2_O_2_ through hydrogen-bond-induced end-bridge adsorption and decomposing it into O_2_ (Figure 10).

#### 3.2.2 Converting ROS to toxic radicals

In recent years, efforts have been made to design catalysts that can specifically bind to ROS (such as H_2_O_2_) produced by the human body Table 1. These catalysts can convert adsorbed H_2_O_2_ into radicals, thereby selectively killing tumor cells named as chemodynamic therapy (CDT). In the acidic tumor microenvironment (TME), H_2_O_2_ is often overexpressed. Efficiently converting it into OH (Yang et al., 2023; Liu et al., 2024; Xu et al., 2024), O_2_^−^ (Cai et al., 2022) or O^2−^ (Wang et al., 2022a) that selectively kill tumor cells without causing off-target toxicity to normal tissues and organs holds promise for clinical anti-tumor therapy. It is known that transition metals such as Fe, Cu, and Co have suitable adsorption energies for H_2_O_2_. Therefore, current research focuses on designing various ligands to guide the charge redistribution of the central atom to facilitate electron transfer with H_2_O_2_ at the microscopic level.

**TABLE 1:** Types and tumor inhibition rates of different single-atom nanozymes.

Materials	Metal atoms	Supports	Mimic function	Tumor inhibition rate	Ref.
FeN_5_ SAzyme	Fe	ZIF-8	POD-like	Intravenous (i.v.) injection: 78.0%	Xu et al. (2022)
				Intratumoral (i.t.) injection: 91.9%	
Fe-Ds/ABTS@HA	Fe	CDs	POD-like	58.4% (+laser)	Yang et al. (2023)
Cu-N/S-C SAzymes	Cu	ZIF-8	POD-like	85.9% (+NIR)	Xu et al. (2024)
Co-SAs@NC	Co	2-methylimidazole	OXD-like	66%; 92% (+DOX)	Cai et al. (2022)

**FIGURE 11:**
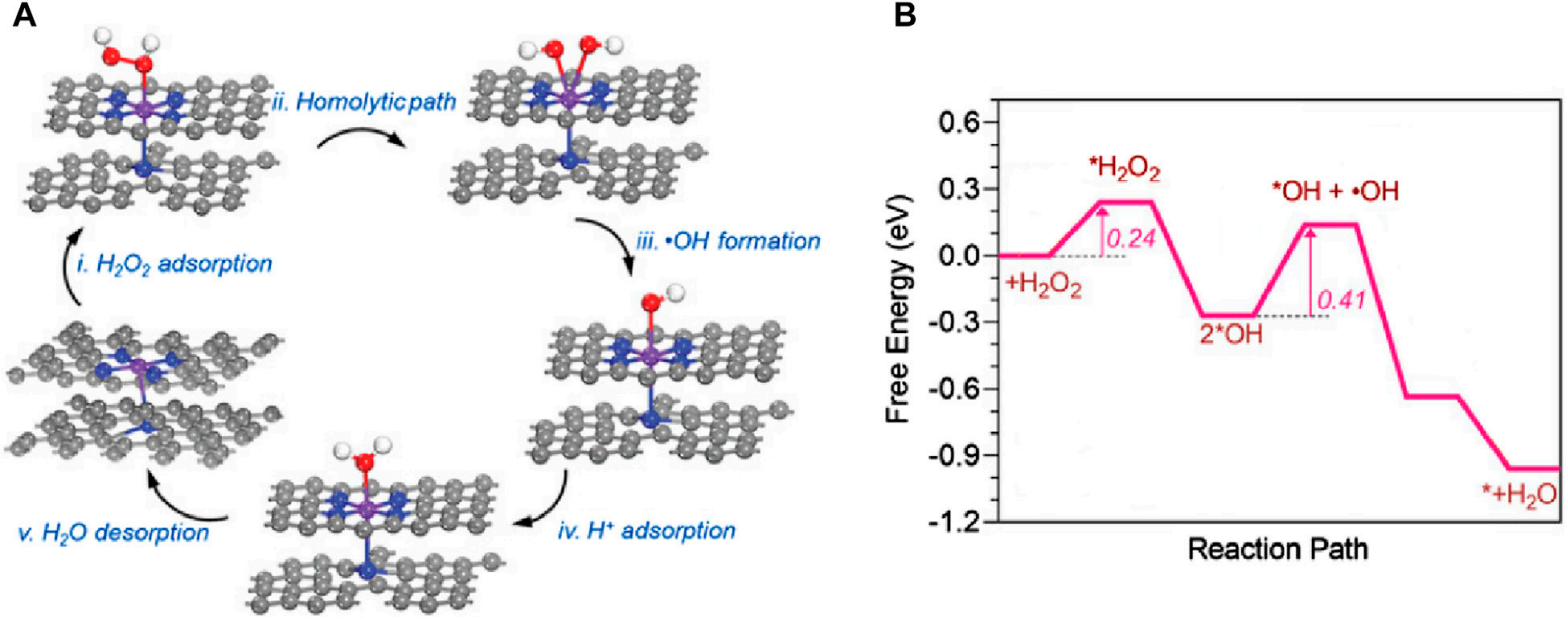
(Xu et al., 2022) **(A)** The catalytic mechanism of peroxidase-like reactions on FeN_5_ **(B)** The corresponding free energy of FeN_5_ peroxidase-like reactions.

**FIGURE 12:**
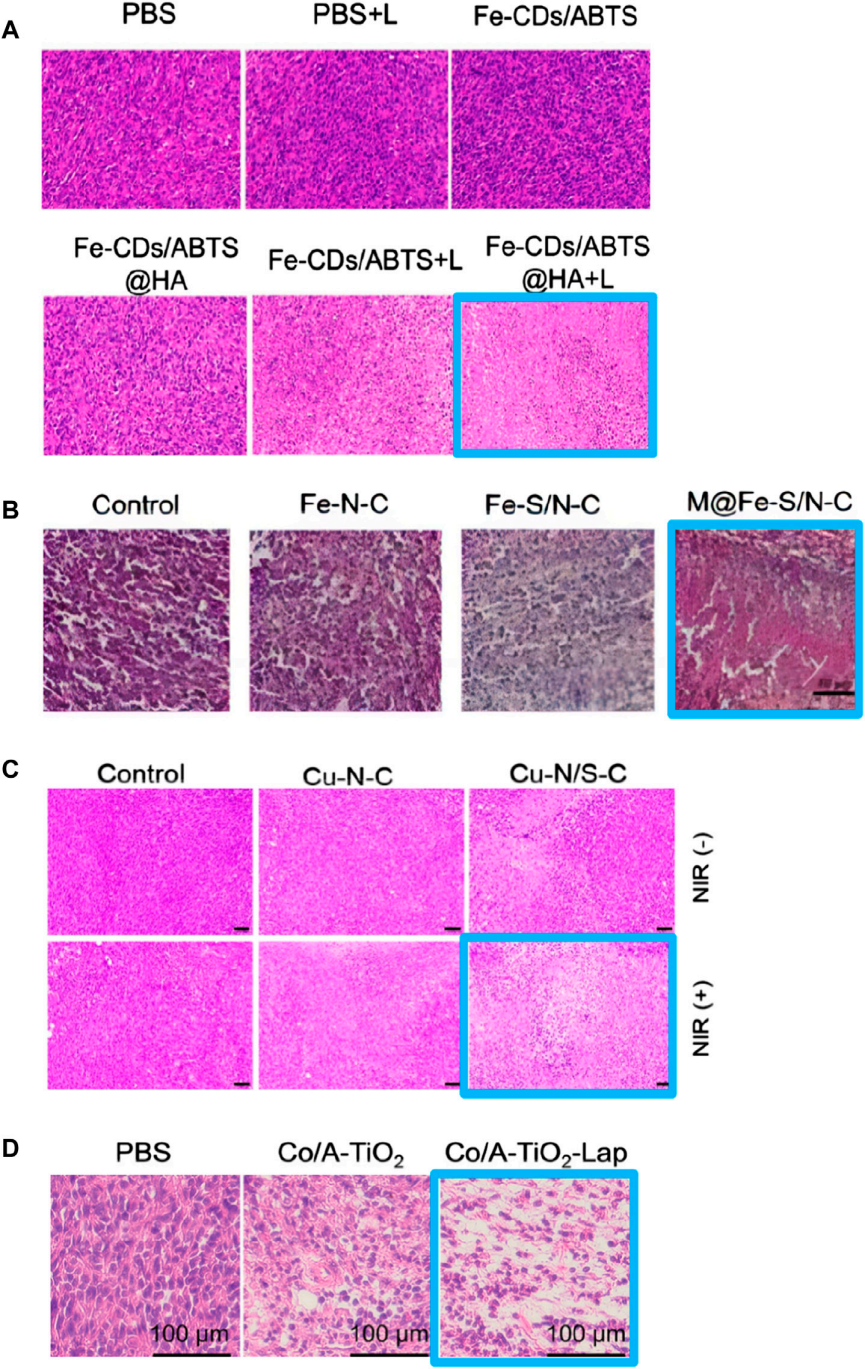
**(A)** (Yang et al., 2023) **(B)** (Liu et al., 2024) **(C)** (Xu et al., 2024) **(D)** (Wang et al., 2023a) H&E staining of tumor tissues after treatment with different single-atom nanozymes.

Metal centers such as Fe and Cu exhibit advantages in demonstrating peroxidase (POD)-like enzyme activity. Researchers utilize various ligands to regulate their spatial structure, making them more efficient in adsorbing H_2_O_2_ and converting it into OH. In 2022, Xu et al. (2022) (Figure 11) designed an FeN_5_ SAzyme with a five-coordinate structure that can effectively adsorb H_2_O_2_. Biological tests indicated that it has a low hemolysis rate and minimal damage to the major organs of mice, suggesting good *in vivo* biocompatibility. Furthermore, it exhibited high tumor inhibition rates, with 78.0% inhibition via intravenous injection and 91.9% inhibition via intratumoral injection, indicating its potential as an efficient anti-tumor agent. In 2023, Yang et al. (2023) designed a non-heme iron SAzyme (Fe-CDs/ABTS@HA), featuring a distorted and non-planar Fe-O_3_N_2_ active site. This twisted five-coordination geometry allows the single iron site to become more saturated, keeping the adsorption energy within an optimal range. This enables better adsorption and catalytic conversion of ROS into OH and more efficient desorption of the produced H_2_O. Vivo and vitro studies have demonstrated that Fe-CDs/ABTS@HA can induce tumor necrosis in mice. Under laser irradiation, the cell apoptosis rate and tumor growth inhibition rate reached 96.6% and 58.4%, respectively. Hematoxylin and eosin (H&E) staining of tumor tissues (Figure 12A) revealed more pronounced necrosis and pyknosis with Fe-CDs/ABTS@HA under laser irradiation. Additionally, Fe-CDs/ABTS@HA exhibited low toxicity to other mouse tissues, indicating high tissue compatibility. In 2023, Liu et al. (2024) utilized sulfur atom doping to disrupt the symmetrical charge distribution around the Fe metal center, designing iron-based SAzymes (Fe-S/N-C SAzymes) with an asymmetric charge distribution. By coating the Fe-S/N-C SAzymes with macrophage membranes, they achieved M@Fe-S/N-C with a smoother surface. The Fe sites in this catalyst exhibit enhanced adsorption capacity and electron transfer ability, leading to improved catalytic activity. Consequently, it can more effectively adsorb H_2_O_2_ and convert it into OH. H&E staining of tumor tissues (Figure 12B) revealed significant nuclear shrinkage and fragmentation in the M@Fe-S/N-C group, indicating that M@Fe-S/N-C effectively disrupted the tumor. Treatment of mice with M@Fe-S/N-C did not affect their body weight, demonstrating high biocompatibility. Similarly, in 2024, Xu et al. (2024) employed sulfur engineering technology to design a copper-based SAzyme (Cu-N/S-C SAzymes), incorporating sulfur atoms into the catalyst. This incorporation resulted in the redistribution of electrons around the copper atoms, leading to an asymmetric distribution. The introduction of sulfur atoms enhanced the electron transfer process between the Cu-N_1_S_2_ active center and H_2_O_2_, facilitating the adsorption and activation of H_2_O_2_, thereby more effectively generating OH. Under near-infrared (NIR) light irradiation, Cu-N/S-C SAzymes achieved a tumor inhibition rate of 85.9%. H&E staining of tumor issues after phototherapy (Figure 12C) revealed a significant decrease in the proportion of purple-stained nuclei, indicating high tumor inhibition rates. Biochemical analysis of the blood from other tissues demonstrated high biosafety, confirming its potential as an efficient and low-toxicity tumor therapeutic agent.

The Co metal center exhibits advantages in demonstrating oxidase (OXD)-like enzyme activity. Researchers utilize various ligands to regulate its spatial structure, enhancing its ability to adsorb H_2_O_2_ and convert it into O_2_^−^ or O^2−^.

Based on the high catalytic efficiency exhibited by cobalt in various catalytic reactions and the efficient exposure of active sites on porous carbon with a large surface, Cai et al. (2022) constructed Co-SAs@NC SAzymes with cobalt highly dispersed on nitrogen-doped porous carbon. The Co sites can effectively adsorb H_2_O_2_ and convert it into O_2_, thereby alleviating hypoxia in cancerous tissue (Datta et al., 2021). Furthermore, these Co sites can continuously catalyze the conversion of O_2_ into highly cytotoxic O_2_^−^, effectively killing tumor cells. Vivo studies showed that the injection of Co-SAs@NC alone achieved a tumor inhibition efficiency of 66% while the tumor inhibition efficiency increased to 92% when combined with doxorubicin (DOX).

In 2022, Wang et al. (2022a) used TiO_2_ as a reducible support to prepare Co/A-TiO_2_ SAzymes with low Co^2+^/Co^3+^ ratios by modifying the local coordination environment of Co metal centers through strong electronic metal-support interactions between atomic metals and the surrounding support (Yang et al., 2020). Theoretical studies indicate that the lower the charge on atomic Co, the higher its activity in catalyzing the decomposition of H_2_O_2_ into O_2_. Consequently, Co/A-TiO_2_ SAzymes exhibit high tumor treatment efficacy. Co/A-TiO_2_ SAzymes can selectively convert H_2_O_2_ into O_2_, potentially alleviating hypoxia in tumor regions, and promote the generation of ROS. These ROS can further act as substrates in OXD-like enzymatic reactions, generating O^2−^ which are more toxic to tumor cells than OH. The catalytic performance of Co/A-TiO_2_ SAzymes was also validated by ROS indicator staining of tumor cells. H&E staining of tumor tissues (Figure 12D) revealed that the combination of Co/A-TiO_2_ SAzymes with β-Lapachone (Lap), which accelerates ROS generation by promoting lactate accumulation, resulted in severe necrosis in tumor tissues, demonstrating the high tumor inhibition rate of this SAzyme.

#### 3.2.3 Multi-enzymatic cascade reactions

In biological systems, biocatalytic cascade reactions occur within organelles, which are separated by cell membranes to prevent interference between reactions, thereby enhancing production efficiency. This strategy has garnered significant attention in the burgeoning field of systems chemistry. Unlike synergistic catalysis, where multiple catalysts operate simultaneously in the same step or reaction (Zhu et al., 2022), biocatalytic cascades involve a sequential, step-by-step catalytic process. Metal-organic frameworks (MOFs) are employed to create distinct spatial compartments that facilitate multi-enzyme cascade reactions. This methodology is particularly applied in tumor therapy to enhance therapeutic outcomes using SAzymes (Man et al., 2022). For instance, Cai et al. (2022) utilized N-doped porous carbon within MOFs to synthesize Co-SAs@NC. This composite acts as a catalase (CAT) mimic to convert hydrogen peroxide (H_2_O_2_) into oxygen (O_2_), and as an oxidase (OXD) mimic to transform O_2_ into highly cytotoxic superoxide (O_2_^−^) radicals.

## 4 Conclusion

The emergence of SAzymes has changed people’s long-term understanding of the application of inorganic materials in the biomedical field, making them aware that inorganic nanomaterials may also play an important role in this field. Traditional enzymes are essentially organic macromolecules such as proteins or RNA, which are often not heat-resistant and have a narrow range of pH values. Therefore, they have the characteristics of being difficult to extract, preserve, and easily inactivated in practical applications; The SAzyme, as an inorganic material, compensates for this drawback.

Compared to the complex and intricate three-dimensional spatial structure of natural enzymes, the active center of SAzymes is only constructed by a single atom, lacking strict geometric and electronic structure constraints, resulting in lower substrate specificity and selectivity. In order to improve the catalytic specificity of SAzymes and make them more widely used in the field of biocatalysis, the current main methods are to change the coordination situation of the central atom (Xie et al., 2020; Wang et al., 2023a), change the charge distribution state of the central atom (Chen et al., 2023b; Liu et al., 2024), design defects (Wang et al., 2023a), or regulate their morphology characteristics (Yang et al., 2023) to endow them higher substrate selection and adsorption ability, thereby improving their catalytic specificity.

SAzymes utilize single atoms as active centers, thus avoiding the problem of molecular-level biomolecule inactivation. These single-atom catalysts have high atomic dispersion and large specific surface area, which theoretically endows them with superior catalytic activity. Nevertheless, SAzymes still face some limitations, and we propose several promising solutions to address these challenges:

(1) At present, research on SAzymes is mostly focused on the field of biomedicine, especially in areas such as anti-tumor, antibacterial, and antioxidant properties. However, it remains to be debated whether SAzymes are harmful to the human body as they are prepared from materials such as metals (Wang et al., 2015). Recently, most studies have been conducted on its physiological stability and biosafety (but based on whether it causes harm to other tissues and organs). However, there is still relatively little research on whether it will bring side effects and whether it has long-term safety. Therefore, although SAzymes exhibit efficient catalytic performance and good stability, their current applicability to the human body remains to be studied and cannot be directly applied in clinical treatment.
(2) The existing SAzyme materials are mainly based on non-precious metal elements (such as Co, Cu, Fe, etc.), while the exploration of some precious metals and rare metal elements with excellent performance in the field of chemical catalysis is still relatively lagging behind. Therefore, expanding the types of SAzymes, especially the inclusion of precious metals and rare metal elements, is expected to endow SAzymes with more excellent catalytic activity and novel functions, thereby promoting the better application prospects of SAzymes in the field of oncology.
(3) There is a lack of a unified database to evaluate the different possible active intermediates and corresponding activation energies of SAzymes with different metal bases catalyzing a unified substrate. Therefore, it is difficult to select a certain kind of SAzyme having certain metal center with excellent catalytic performance, much less regulate the coordination environment. Therefore, a Materials Genome Initiative is urgently needed in the field of SAzymes.
(4) SAzymes have undergone the evolution of type selection (metal oxide type, metal organic type or polypeptide protein aggregate type) to the regulation of the surrounding environment of the central atom, and then to the current combination of other common cancer treatment methods (combined with photothermal therapy or other anti-tumor drugs). This synergistic effect in combination with other common cancer treatments contributes to the achievement of better tumor outcomes with SAzymes. However, the side effects of the beam on the human body (e.g., the burning effect on the human body) should be considered when applying photothermal therapy, and the drug interaction should be considered when using multiple antineoplastic drugs.
